# Successful Percutaneous Retrieval of Embolized Septal Occluder Device from Aortic Arch and Placement of a Newer Septal Occluder Device in Combined Procedure

**DOI:** 10.1155/2016/1032801

**Published:** 2016-12-27

**Authors:** Natraj Katta, Sandeep Gautam, Richard Webel

**Affiliations:** Division of Cardiovascular Medicine, University of Missouri School of Medicine, Columbia, MO, USA

## Abstract

Embolization of the Amplatzer Septal Occluder (ASO) device (St. Jude Medical, Minnesota) after percutaneous closure of atrial septal defect (ASD) is a rare and potentially catastrophic complication. Percutaneous retrieval of the embolized device is gaining ground as an acceptable method, although these patients are usually subsequently referred for open surgical closure of the ASD. We present a unique case of percutaneous retrieval embolized ASO device and placement of newer larger ASO device in a single procedure.

## 1. Case Report

**Figure 1:**
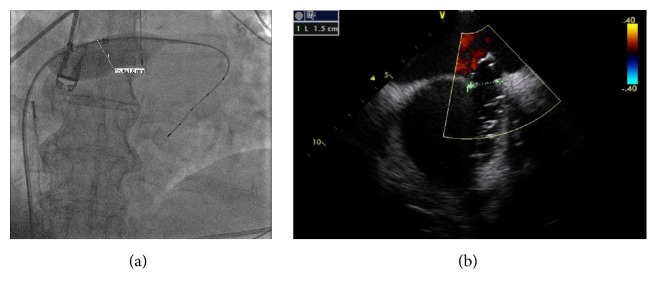
(a) Sizing balloon showing the diameter of the ASD. (b) Intracardiac echocardiogram showing the diameter of the ASD.

**Figure 2:**
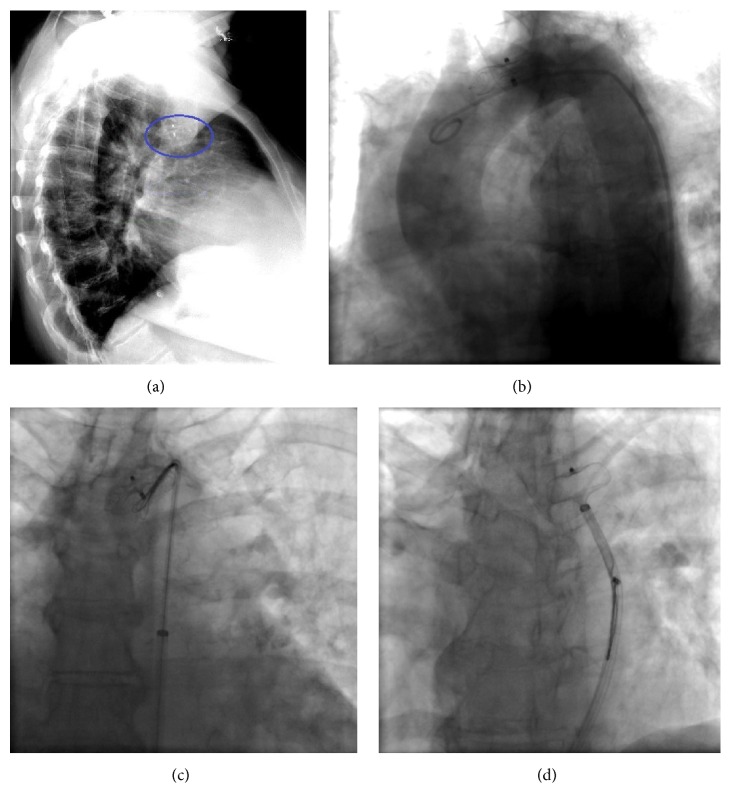
(a) Chest radiograph, lateral view showing the embolized ASO. (b) Aortic arch angiography using angled pigtail catheter. (c) Using En-Snare, capturing of the metallic tip of the right atrial disc of the ASO. (d) Retraction of the entire ASO into the long cook sheath.

**Figure 3:**
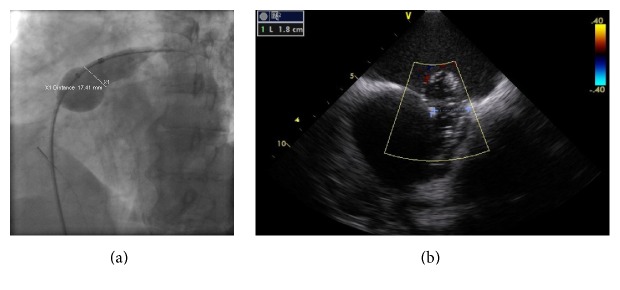
(a) Resizing of the ASD using the sizing balloon. (b) Resizing of the ASD using the intracardiac echocardiogram.

A 75-year-old female patient with history of persistent atrial fibrillation after multiple failed cardioversions and septum secundum type of ASD presented with worsening exertional dyspnea of six-month duration. She was previously intolerant to amiodarone. She underwent elective catheter based atrial fibrillation ablation followed by closure of the ASD during a combined percutaneous procedure. ASD closure was performed using a 16 mm Amplatzer Septal Occluder (ASO) device after measuring the diameter of the defect at 15.4 ± 1.0 mm (Figure 1(a)) with sizing balloon and 15 mm (Figure 1(b)) with intracardiac echocardiogram (ICE). After the deployment of the device, ICE showed minimal residual shunt flow confirming correct position. The following day, a transthoracic echocardiogram (TTE) revealed severe right to left shunt indicating recurrence of ASD, without visualization of the ASO device. A chest radiograph (Figure 2(a)) showed the embolized ASO device in the aortic arch. Patient had no new symptoms except recurrence of atrial fibrillation requiring cardioversion. Patient was taken to cardiac catheterization laboratory for possible percutaneous retrieval of the embolized ASO device and placement of a newer device. 6 F × 11 cm sheath was placed in right femoral artery. Aortic arch angiography was performed with a 5 F × 110 cm pigtail catheter (Figure 2(b)). 6 F sheath was then exchanged with a 10 F × 80 cm long cook sheath (Cook Medical Inc. Indiana). Using 6 F 12–20 mm × 120 cm En-Snare System (Merit Medical Systems, Inc., Utah) through a 6 F Hockey Stick II guiding catheter (Medtronic Inc., Minnesota), we were able to snare the metallic tip on the right atrial disc of ASO device (Figure 2(c)). Subsequently, entire device was collapsed into the long sheath which was safely removed (Figure 2(d)). Thereafter, a 20 mm ASO device placed using a 9 F Amplatzer delivery system (St. Jude Medical, Minnesota), after resizing of the defect. This time the sizing balloon yielded diameter of 17.41 mm (Figure 3(a)) and ICE of 18 mm (Figure 3(b)). Both ICE and TTE showed properly positioned ASO device with minimal residual shunt. No procedure related complications occurred. Patient was discharged 2 days later.

## 2. Discussion

The incidence of occluder device embolization after ASD closure is 0.55%–1.4% [1, 2]. Nearly half of the embolized ASO devices tend to lodge in the left atrium and aorta [3]. According to the MAUDE (Manufacturer and User Facility Device Experience) database, only a minority (16.7%) of embolized devices are retrieved using percutaneous approach [2]. Undersized device, floppy or inadequate rim, device malposition, and excessive tension on the delivery cable during the deployment are the possible mechanisms for device embolization [2]. In our case, we believe that undersizing was the possible mechanism, which in turn was probably due to noncircular shape of the defect. In previous case reports, Gooseneck type of snare system was used [4–6], whereas we are able to retrieve the device using the En-Snare System, showing that there are various options in choosing the retrieval system, although merits of one system over the others are unclear. According to a survey of the ASO company-designated proctors, approximately half of the patients had percutaneous closure of the ASD after the embolized device retrieval, but it is not reported whether they were done as a combined procedure or at a later date [7]. To the best of our knowledge, our case is the first reported case of percutaneous retrieval of embolized device and percutaneous placement of newer device performed as combined procedure. We also performed atrial fibrillation ablation with ASD closure on the same day. As arrhythmia control is difficult to achieve with ASD closure alone in patients with atrial fibrillation and technical challenges associated with trans-septal access after device closure, it is considered appropriate to perform atrial fibrillation ablation prior to ASD closure [8, 9].

## 3. Conclusion

Percutaneous retrieval of the embolized ASO device is still not widely performed. Also, there is no consensus regarding the appropriate timing of the reclosure of the ASD percutaneously after the retrieval of embolized device. With proper planning, percutaneous retrieval and placement of newer device can be safely performed in single setting. We also believe that, in patients with atrial fibrillation and atrial septal defect, it is reasonable to perform atrial fibrillation ablation prior to ASD closure.
